# Influence of electronically conductive additives on the cycling performance of argyrodite-based all-solid-state batteries†

**DOI:** 10.1039/c9ra10253a

**Published:** 2020-01-07

**Authors:** Florian Strauss, Dominik Stepien, Julia Maibach, Lukas Pfaffmann, Sylvio Indris, Pascal Hartmann, Torsten Brezesinski

**Affiliations:** Battery and Electrochemistry Laboratory, Institute of Nanotechnology, Karlsruhe Institute of Technology (KIT) Hermann-von-Helmholtz-Platz 1, 76344 Eggenstein-Leopoldshafen Germany florian.strauss@kit.edu torsten.brezesinski@kit.edu; Institute for Applied Materials-Energy Storage Systems (IAM-ESS), Karlsruhe Institute of Technology (KIT) Hermann-von-Helmholtz-Platz 1, 76344 Eggenstein-Leopoldshafen Germany; Karlsruhe Nano Micro Facility (KNMF), Karlsruhe Institute of Technology (KIT) Hermann-von-Helmholtz-Platz 1, 76344 Eggenstein-Leopoldshafen Germany; Helmholtz Institute Ulm (HIU) Electrochemical Energy Storage Helmholtzstr. 11 89081 Ulm Germany; BASF SE Carl-Bosch-Str. 38 67056 Ludwigshafen Germany

## Abstract

All-solid-state batteries (SSBs) are attracting widespread attention as next-generation energy storage devices, potentially offering increased power and energy densities and better safety than liquid electrolyte-based Li-ion batteries. Significant research efforts are currently underway to develop stable and high-performance bulk-type SSB cells by optimizing the cathode microstructure and composition, among others. Electronically conductive additives in the positive electrode may have a positive or negative impact on cyclability. Herein, it is shown that for high-loading (pelletized) SSB cells using both a size- and surface-tailored Ni-rich layered oxide cathode material and a lithium thiophosphate solid electrolyte, the cycling performance is best when low-surface-area carbon black is introduced.

## Abstract 2

Low-surface-area carbon black helps to improve the performance of bulk-type all-solid-state batteries using NCM622 cathode material and argyrodite Li_6_PS_5_Cl solid electrolyte.

## Graphical abstract

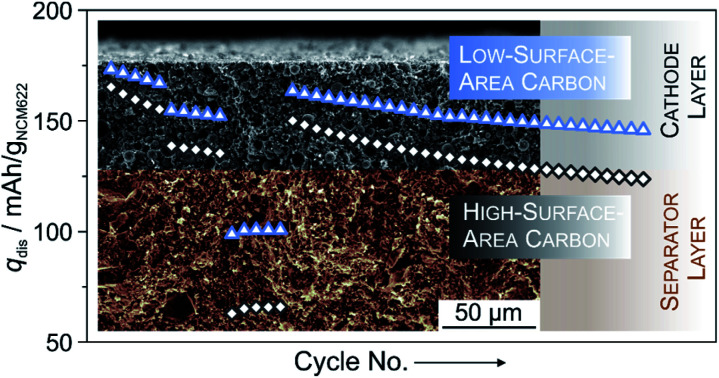

## Introduction

All-solid-state batteries (SSBs) are widely considered a serious contender to replace conventional Li-ion technology, potentially offering improved power and energy densities.^1^ However, several barriers must be overcome before market entry can be achieved, some of which relate to interfacial issues between the active and inactive electrode constituents and the solid electrolyte (SE) that adversely affect the cycling performance and stability.^2,3^ At the positive electrode side, detrimental side reactions can be somewhat suppressed by applying a protective surface coating to the cathode active material (CAM), such as LiNbO_3_.^4–7^

In general, cathode composites for SSBs must offer sufficient percolation pathways for effective electronic and lithium ion transport.^8–10^ On the one hand, the transport properties can be improved by using highly conductive SEs showing negligible grain boundary resistance (especially for processing by cold pressing), with lithium thiophosphates being the most promising materials at present.^11,12^ On the other hand, they can be altered by tailoring the CAM secondary particle size, as shown recently for cathode composites of Li_1+*x*_(Ni_1−*y*−*z*_Co_*y*_Mn_*z*_)_1−*x*_O_2_ (NCM) and β-Li_3_PS_4_.^10^

The purpose of this study is to examine the possibility of further increasing the cycling performance of pelletized SSB cells by addition of an electronically conductive additive (1 wt%) to the cathode composite layer. In particular, three carbons of different specific surface area and morphology, namely Super C65, Ketjenblack (KB) and carbon nanofibers (CNF) with *S*_BET_ ≈ 65, 1400 and 24 m^2^ g^−1^, respectively, as well as titanium carbide (TiC) nanopowder have been tested and the results compared to the cyclability of additive-free cathode composite containing LiNbO_3_-coated NCM622 (60% Ni) CAM and argyrodite Li_6_PS_5_Cl SE. Differences in capacity fading can be linked directly to differences in interfacial degradation, as revealed by electrochemical impedance spectroscopy and X-ray photoelectron spectroscopy. The motivation to study TiC as an additive emerged from the fact that it exhibits both a reasonably high electronic conductivity and a higher oxidative stability than carbonaceous materials.^13–16^

## Materials and methods

### Materials

Small particle size NCM622 [Li_1+*x*_(Ni_0.6_Co_0.2_Mn_0.2_)_1−*x*_O_2_] (*d*_50_ = 2.9 μm, *d*_90_ = 6.0 μm) was supplied by BASF SE.^10,17^ Prior to use, a 1 wt% LiNbO_3_ coating was applied to the cathode material.^4,5^ Super C65 carbon black (Timcal), Ketjenblack EC-600JD (AkzoNobel), conical carbon nanofibers (100 nm × 20–200 μm; Sigma Aldrich) and TiC nanopowder (<200 nm; Sigma Aldrich) were used as electronically conductive additives. All materials were dried at 300 °C overnight in a vacuum and then stored in an Ar-filled glovebox ([O_2_] and [H_2_O] < 0.1 ppm; MBraun).

Li_6_PS_5_Cl solid electrolyte was synthesized by high-energy milling of a mixture of Li_2_S (99.9%; Sigma Aldrich), P_2_S_5_ (99%; Sigma Aldrich) and LiCl (>99%; Alfa Aesar), with the former being used in a less than stoichiometric amount (by 10 mol%) and the latter being pre-dried in a vacuum. Finally, the recovered powder was heated at 300 °C for 5 h in a vacuum.

### Electrode preparation

Cathode composite was prepared by mixing NCM622 and Li_6_PS_5_Cl in a 7 : 3 ratio by weight plus 1 wt% conductive additive at 140 rpm for 30 min under an Ar atmosphere in a planetary ball mill (Fritsch) using 10 zirconia balls of diameter 10 mm. Anode composite was prepared by following the same protocol, but using a mixture of carbon-coated Li_4_Ti_5_O_12_ (NEI Corporation), Super C65 carbon black and Li_6_PS_5_Cl in a 3 : 1 : 6 weight ratio.

### Cell assembly and electrochemical measurements

A customized setup comprising two stainless steel dies and a PEEK sleeve was utilized in the electrochemical testing of the SSB cells. For the preparation of circular pellets of 10 mm diameter, Li_6_PS_5_Cl (100 mg) was compressed first at a pressure of 125 MPa. Then, cathode composite (11 mg) was pressed at 375 MPa onto the solid electrolyte, followed by anode composite (65 mg) onto the other side. Galvanostatic measurements were performed at 25 °C and at a C/10 rate (1C = 180 mA g_NCM_^−1^) between 1.35 and 2.85 V with respect to Li_4_Ti_5_O_12_/Li_7_Ti_5_O_12_ using a MACCOR battery cycler. Prior to charge, the cells were kept at an open-circuit voltage (OCV) for 1 h. Note that a pressure of 55 MPa was maintained during the measurements.

Electrochemical impedance spectroscopy (EIS) was performed using a VMP3 multichannel potentiostat (BioLogic). The spectra were acquired in the frequency range between 100 mHz and 7 MHz with an AC voltage amplitude of 10 mV at OCV (in the discharged state) and fitted using an equivalent circuit with three (*RQ*) elements in series.^18–20^

### X-ray photoelectron spectroscopy (XPS)

The SSB cells were disassembled in an Ar-filled glovebox. Parts of the cathode layer were mounted onto a sample holder using conductive Cu tape and then transferred under inert conditions to the XPS chamber. XPS was performed using a K-Alpha XP spectrometer (Thermo Fisher Scientific) with monochromatic Al-Kα radiation (400 μm spot size). The kinetic energy of photoelectrons was measured using a 180° hemispherical energy analyzer at a constant pass energy of 50 eV. Data acquisition and processing were performed using Thermo Avantage software. The spectra were fitted with one or more Voigt profiles and by use of a smart background function. All data are referenced to the C 1s (C–C, C–H) binding energy at 285.0 eV (Fig. S1†).

### Scanning electron microscopy (SEM)

Imaging and mapping data were collected on a MERLIN scanning electron microscope (Carl Zeiss) at an acceleration voltage of 30 kV and probe current of 20 nA. The SSB cells were disassembled in an Ar-filled glovebox and the pellets mounted onto a sample holder using conductive Cu tape to reduce the carbon signal for energy dispersive X-ray spectroscopy (EDS) mapping. Sample transfer under inert conditions to the microscope was done using a customized holder.

### X-ray diffraction (XRD)

The phase composition of the Li_6_PS_5_Cl solid electrolyte was examined in borosilicate glass capillaries (0.48 mm inner diameter, 0.01 mm wall thickness; Hilgenberg) by powder XRD using a STADI P diffractometer (STOE) equipped with a Cu-Kα radiation source (curved Ge (111) monochromator) and a MYTHEN 1K detector (DECTRIS). XRD patterns were collected in moving PSD mode in the range between 10 and 70° 2*θ* with a step size of 0.012° and an exposure time of 6 s per step. Rietveld analysis was done using TOPAS-Academic V5 software.

## Results and discussion

**Fig. 1:**
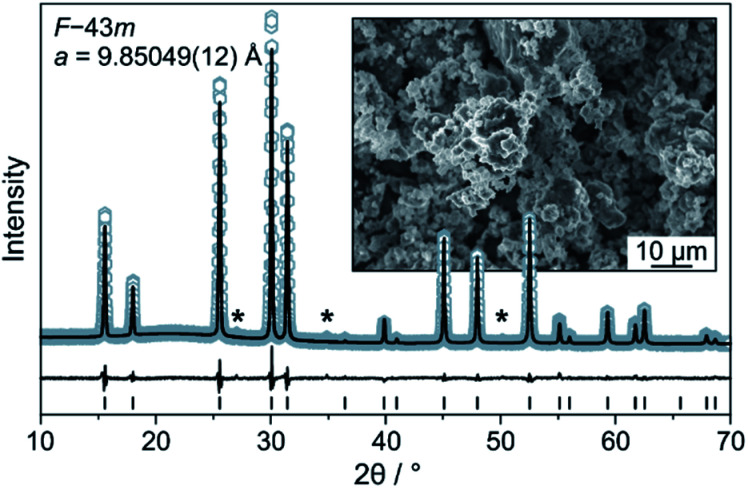
Rietveld analysis of the XRD pattern for the Li_6_PS_5_Cl solid electrolyte. Asterisks denote impurities. The inset is a low-magnification SEM image of the as-prepared solid electrolyte powder.

The Li_6_PS_5_Cl SE was prepared by high-energy milling and subsequent heating at 300 °C in a vacuum. The experimental XRD pattern and the corresponding Rietveld plot (*R*_wp_ = 6.4, GoF = 1.5) are shown in Fig. 1. The value of the refined *a* lattice parameter is in good agreement with that of Deiseroth *et al.* for argyrodite Li_6_PS_5_Cl prepared at 550 °C [9.859(2) Å].^21^ SEM imaging showed that the as-synthesized powder consists of particles of various shapes and non-uniform size (inset of Fig. 1). The ionic conductivity of cold-pressed pellets was determined by EIS to be 1.8 mS cm^−1^ at 250 MPa (Fig. S2†) despite the presence of small impurities, such as Li_2_S.

**Fig. 2:**
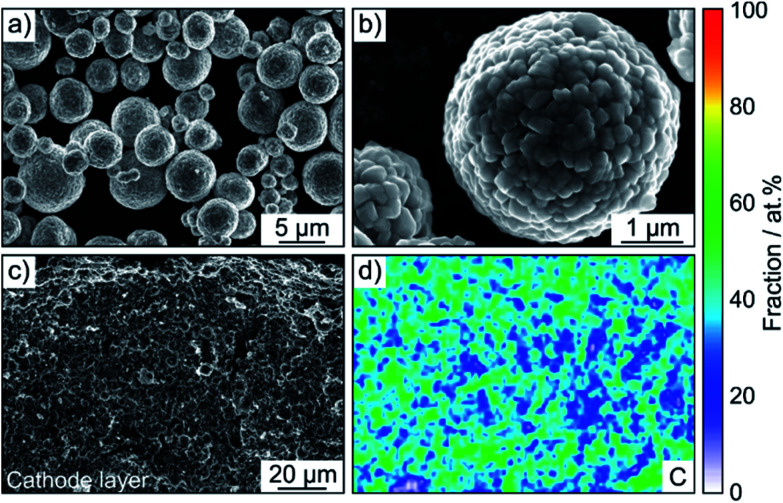
(a and b) Top-view SEM images at different magnifications of the LiNbO_3_-coated NCM622 secondary particles. (c) Low-magnification cross-sectional SEM image of a pristine SSB cathode using Super C65 as conductive additive and (d) the corresponding elemental map of carbon.

A LiNbO_3_ surface coating was applied to the NCM622 to prevent side reactions (as much as possible) at the interface of CAM and SE from occurring during cycling operation. EDS mapping confirmed the presence of niobium (Fig. S3†). In addition, the SEM images in Fig. 2a and b indicate that the coating has no immediate impact on the primary and secondary particle morphology (the top surface appears to be relatively smooth). Moreover, as reported recently, protective surface coatings containing (lithium) carbonate species are capable of considerably improving the overall performance of SSB cathodes.^7,22^ In the present work, the Li_2_CO_3_ content was quantified by acid titration and found to be 0.3 wt%.^23^

SSB cells made of (high-loading) pellet stacks, with Li_6_PS_5_Cl and Li_4_Ti_5_O_12_ serving as the separator and anode, respectively, were prepared by cold pressing. According to cross-sectional SEM and EDS, the cathode thickness is in the range between 60 and 90 μm and the different electrode constituents are distributed in a uniform manner across the respective layers, as shown in Fig. 2c, d and S4† for pristine pellet stacks using Super C65, KB, CNF and TiC as conductive additive.

**Fig. 3:**
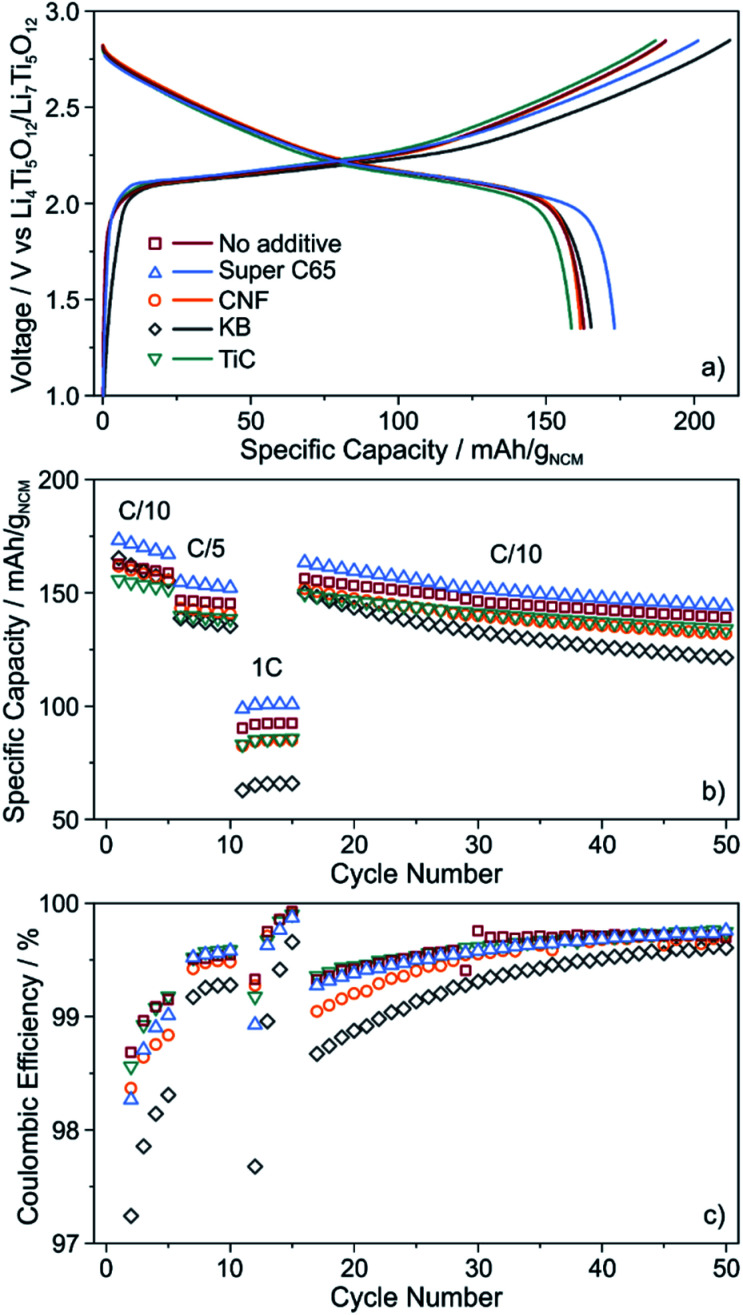
(a) Initial voltage profile, (b) specific discharge capacity and (c) coulombic efficiency of SSB cells with and without conductive additive. All data are averaged from two independent cells.

The cells were cycled at 25 °C in the voltage range from about 2.9 to 4.4 V with respect to Li^+^/Li (1.35–2.85 V *vs.* Li_4_Ti_5_O_12_/Li_7_Ti_5_O_12_). As can be seen from Fig. 3a, the initial voltage profiles at a C/10 rate are typical of Ni-rich layered oxide CAMs, with small but distinct differences in the specific (dis)charge capacity and coulombic efficiency. The cells using Super C65 delivered the largest first cycle discharge capacity of 173 mA h g_NCM_^−1^, with the coulombic efficiency being around 86%. The additive-free cells and those with KB and CNF revealed comparable discharge capacities of 162–165 mA h g_NCM_^−1^. Except for KB, the coulombic efficiency was 85–86%. The larger irreversibility seen for the KB cells (78% coulombic efficiency) hints at more pronounced side reactions during the course of the first cycle, presumably because of SE decomposition. These reactions already occur at relatively low voltages, as indicated by the reduced slope of the charge trace. The lowest initial charge and discharge capacities (159 mA h g_NCM_^−1^) were found for the cells using TiC additive, corresponding to a coulombic efficiency of 85%.

For higher C-rates, a capacity drop was observed, which is similar for all of the cells, except for KB, where especially at a 1C rate the specific discharge capacity was lower by about 20 mA h g_NCM_^−1^ (Fig. 3b). This is indicative of relatively poorer charge transfer kinetics, most probably because of accelerated degradation at the interface between SE and KB (note that the BET surface area of KB is significantly higher than that of the other additives tested in this work). Capacity fading was apparent with further cycling, the capacity retention being around 85% for the additive-free, Super C65 and TiC cells after 50 cycles. In contrast, addition of KB and CNF led to final discharge capacities corresponding to capacity retentions of only 73 and 81%, respectively, thereby indicating faster performance decay. This result is in agreement with the coulombic efficiency, which except for KB and CNF, stabilized above 98% after 4 cycles (Fig. 3c), suggesting the formation of robust interfaces.

**Fig. 4:**
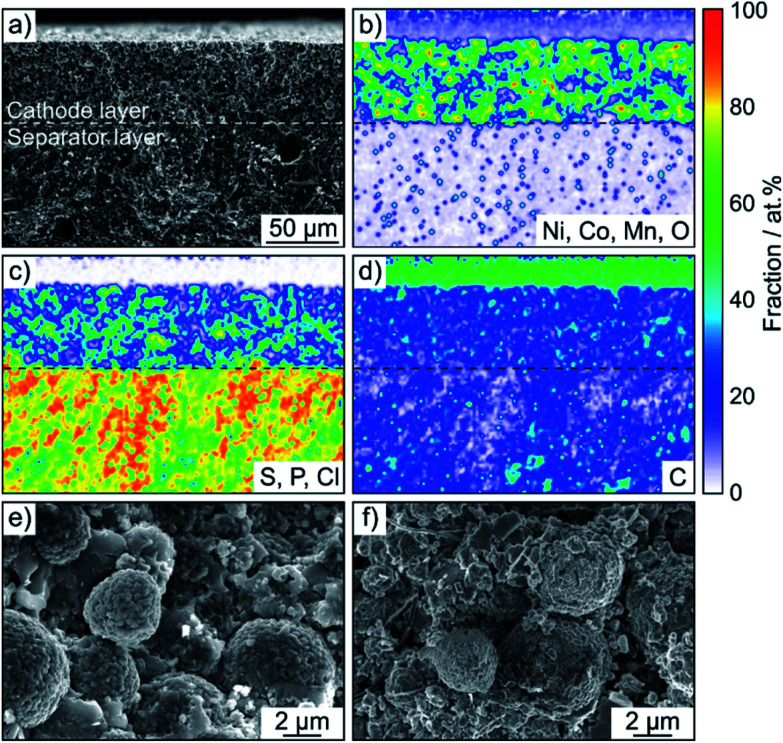
(a) Low-magnification cross-sectional SEM image of a cycled SSB pellet stack using Super C65 as conductive additive and (b–d) the corresponding EDS maps. Only the cathode composite and solid electrolyte separator layers are shown for clarity. (e and f) High-magnification cross-sectional SEM images of cycled SSB cathodes without and with conductive additive (carbon nanofibers), respectively.

SEM was performed to determine if any morphological changes occurred to the SSB pellets upon cycling. To this end, the cells after 50 cycles were disassembled, followed by transfer to the microscope without exposure to laboratory air. As is evident from the cross-sectional images and the corresponding EDS mapping results in Fig. 4 and S5,† the separator and cathode microstructures were preserved quite well and the different electrode constituents were still uniformly distributed. Hence, chemomechanical degradation (*i.e.*, particle fracture, crack formation *etc.*) does not seem to be the primary factor causing the capacity fading.

To gain more insight into the SE degradation, EIS was conducted on the cycled cells. The spectra were fitted using an equivalent circuit previously reported for SSBs (Fig. S6†), (*R*_1_*Q*_1_)(*R*_2_*Q*_2_)(*R*_3_/*Q*_3_)*Q*_4_, with *R*_1_ and *R*_2_ representing the bulk and grain boundary SE resistance, respectively, and *R*_3_ the interfacial SE/cathode (including SE/additive) resistance.^18–20^ For the additive-free cells and those using Super C65 and TiC, *R*_3_ amounted to around 100 Ω. The cells with CNF showed slightly higher interfacial resistance of 120 Ω and for KB, it even increased to 170 Ω. This trend is consistent with the evolution of coulombic efficiency during cycling (Fig. 3c).

**Fig. 5:**
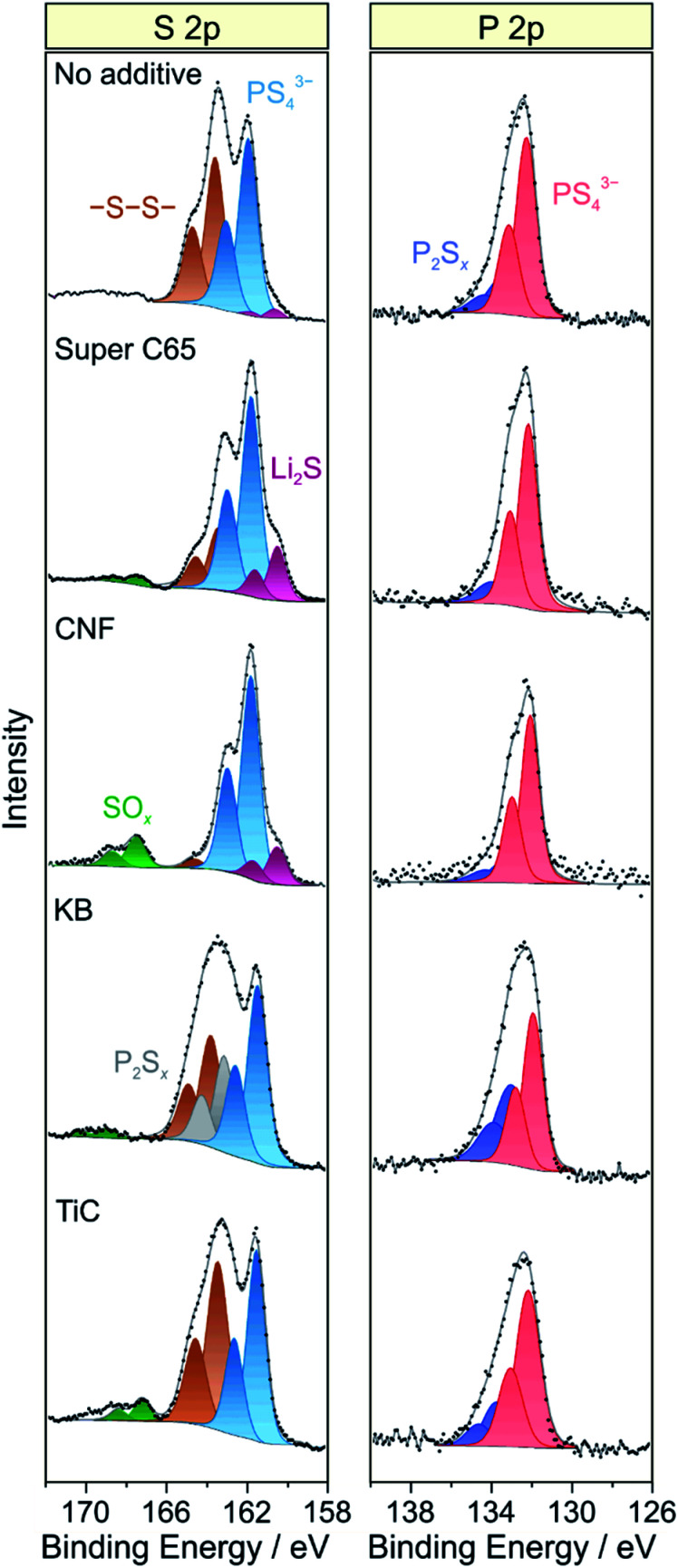
Detailed XPS spectra of the S 2p and P 2p core-level regions of cycled SSB cells with and without conductive additive. The data were acquired from the cathode side of the pellet stacks.

As established in the literature, direct contact between electronically conductive electrode constituents and lithium thiophosphate SEs leads to electrochemical degradation of the latter and consequently to impedance buildup (because of interphase layer formation) and performance decay.^24–27^ To examine the chemical nature of the decomposition products, XPS was conducted on the cathode side of the pellet stacks before and after cycling, with particular emphasis given to the S 2p and P 2p core-level regions. Regarding the S 2p data, the pristine composites showed a single doublet at 161.4–161.5 eV (2p_3/2_), characteristic of the PS_4_^3−^ unit of Li_6_PS_5_Cl (Fig. S7†). Significant changes in S 2p line shape were noticed after 50 cycles (Fig. 5), with appearance of new peaks at higher and/or lower binding energies, providing clear evidence of interfacial SE decomposition. A new doublet was found at 163.6 eV (2p_3/2_), indicative of –S–S– bond formation (polysulfides, elemental sulfur *etc.*). Moreover, in the case of KB, an additional doublet arose at 163.2 eV (2p_3/2_), suggesting the formation of thiophosphate species of general type P_2_S_*x*_ (*x* > 5).^27,28^ The additive-containing cathodes also exhibited the appearance of peaks at binding energies larger than 167 eV because of the presence of SO_*x*_ species.^25,27^ For Super C65, CNF and TiC, the doublet at about 167.5 eV (2p_3/2_) can be assigned to sulfite species. In the case of KB, the doublet was shifted by 1.5 eV to the higher binding energy side, revealing sulfate formation.^27^ From these data, we conclude that the addition of electronically conductive additives either triggers or enhances the formation of oxygenated decomposition products, a hypothesis corroborated by recent studies on thiophosphate-based SSBs with and without carbon additive.^18,25^

At the lower binding energy side, a new doublet at about 160.6 eV (2p_3/2_) was observed when using Super C65, CNF or no additive in the cathode composite, stemming from the formation of Li_2_S.^29,30^ Interestingly, the peaks related to PS_4_^3−^ were shifted by 0.4–0.5 eV to higher binding energies relative to the pristine state when Li_2_S was apparent. This may be due to charging or electronic interface effects during the measurement because of the insulating nature of Li_2_S.^31,32^ Nevertheless, the distinct mechanisms leading to Li_2_S and SO_*x*_ formation are yet to be resolved. We also note that the Ti 2p peaks showed some shift in binding energy after cycling (Fig. S8†), which appears to be indicative of TiO_*x*_C_*y*_ formation, in agreement with data reported in literature.^14,33^

Similar to the S 2p data, a single doublet at 131.8 eV (2p_3/2_) was found in the P 2p spectra of the pristine cathode composites (Fig. S7†). After 50 cycles, the P 2p signal showed clear broadening towards higher binding energies, the new doublet around 133.1 eV (2p_3/2_) being characteristic of species with P–S–P bonding (Fig. 5).^27,28^ Notably, PO_*x*_ formation was prevented from occurring. Such species are usually observed when non-coated oxide CAMs are being used and have been hypothesized to be considerably detrimental to the cyclability of SSB cells.^18,26,27^ Hence, this result underlines the effectiveness of the LiNbO_3_ surface coating. Overall, it can be assumed that the various SE decomposition products identified by XPS do not benefit but rather adversely affect the cell performance. However, defining the effect of individual degradation mechanisms and products on impedance buildup, for example, is very challenging and needs further study.

## Conclusions

In summary, we have studied the influence of electronically conductive additives on the cyclability of pelletized SSB cells using LiNbO_3_-coated NCM622, Li_4_Ti_5_O_12_ and argyrodite Li_6_PS_5_Cl as cathode, anode and solid electrolyte/separator, respectively. Electrochemical testing showed that the addition of 1 wt% Super C65 carbon black to the cathode composite leads to increased capacity and rate performance compared to additive-free cells, virtually without affecting the coulombic efficiency (reversibility). Such cells were still capable of delivering areal capacities of about 1.5 mA h cm^−2^ at a C/10 rate after 50 cycles. In contrast, application of Ketjenblack, carbon nanofibers and titanium carbide was found to worsen the cell cyclability. High-surface-area Ketjenblack, in particular, exerted a strong negative effect on the cycling performance and stability.

XPS analysis revealed that decomposition of the solid electrolyte occurs during cell operation, irrespective of whether or not an additive is being used, despite the presence of a protective surface coating on the NCM622 particles. Apart from that, XPS also showed that the coating is effective in inhibiting the formation of PO_*x*_ species. However, several open questions remain regarding the degradation pathways, the effect of the additive composition or the main functions of the protective coating layer, to name a few, and warrant further examination.

Taken together, our research data demonstrate that the addition of a common carbon black additive may help to enhance the cycling performance of thiophosphate-based bulk-type SSB cells [also when using a high-performance (tailored) cathode material].

## Conflicts of interest

There are no conflicts to declare.

## Supplementary Material

RA-010-C9RA10253A-s001
