# Supplementary material for: Influence of electronically conductive additives on the cycling performance of argyrodite-based all-solid-state batteries
Source: RSC Adv. 2020 Jan 7;10(2):1114–9. doi: 10.1039/c9ra10253a (PMC9046990; doi:10.1039/c9ra10253a)
Supplement: RA-010-C9RA10253A-s001 [file RA-010-C9RA10253A-s001.pdf]

Electronic Supplementary Material (ESI) for RSC Advances.
This journal is © The Royal Society of Chemistry 2019

Electronic Supplementary Information (ESI)

Influence of Electronically Conductive Additives on the Cycling Performance of Argyrodite-Based All-Solid-State Batteries

Florian Strauss,[a,*] Dominik Stepien,[a,b] Julia Maibach,[b,c] Lukas Pfaffmann,[b] Sylvio Indris,[b,d] Pascal Hartmann[a,e] and Torsten Brezesinski[a,*]

[a] Battery and Electrochemistry Laboratory, Institute of Nanotechnology, Karlsruhe Institute of Technology (KIT), Hermann-von-Helmholtz-Platz 1, 76344 Eggenstein-Leopoldshafen, Germany. E-mail: florian.strauss@kit.edu, torsten.brezesinski@kit.edu

[b] Institute for Applied Materials-Energy Storage Systems (IAM-ESS), Karlsruhe Institute of Technology (KIT), Hermann-von-Helmholtz-Platz 1, 76344 Eggenstein-Leopoldshafen, Germany.

[c] Karlsruhe Nano Micro Facility (KNMF), Karlsruhe Institute of Technology (KIT), Hermann-von-Helmholtz-Platz 1, 76344 Eggenstein-Leopoldshafen, Germany.

[d] Helmholtz Institute Ulm (HIU) Electrochemical Energy Storage, Helmholtzstr. 11, 89081 Ulm, Germany.

[e] BASF SE, Carl-Bosch-Str. 38, 67056 Ludwigshafen, Germany.

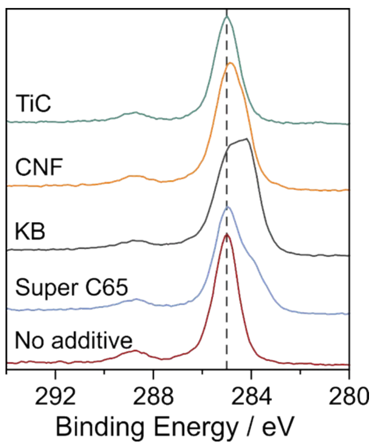

Fig. S1 C 1s core-level spectra for the pristine cathode composites.

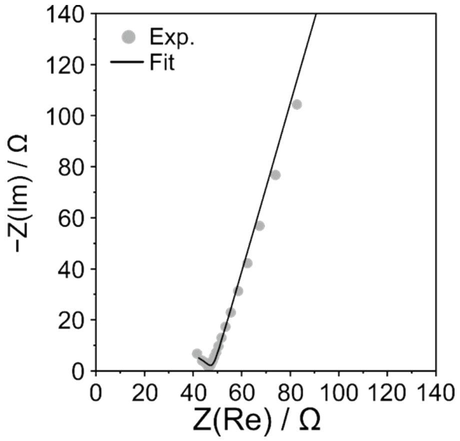

Fig. S2 Room-temperature impedance spectrum for the as-prepared Li_6PS_5Cl solid electrolyte and the corresponding fit to the data.

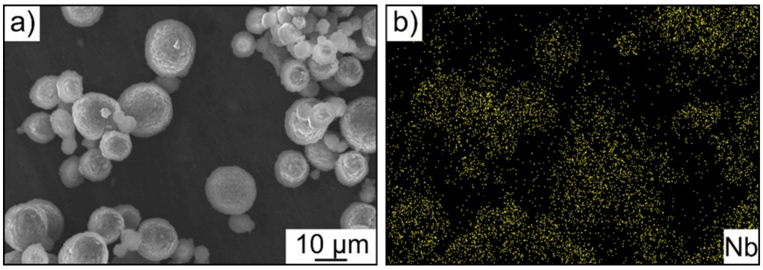

Fig. S3 (a) Top-view SEM image of the $LiNbO_3$-coated NCM622 secondary particles and (b) the corresponding elemental map of niobium.

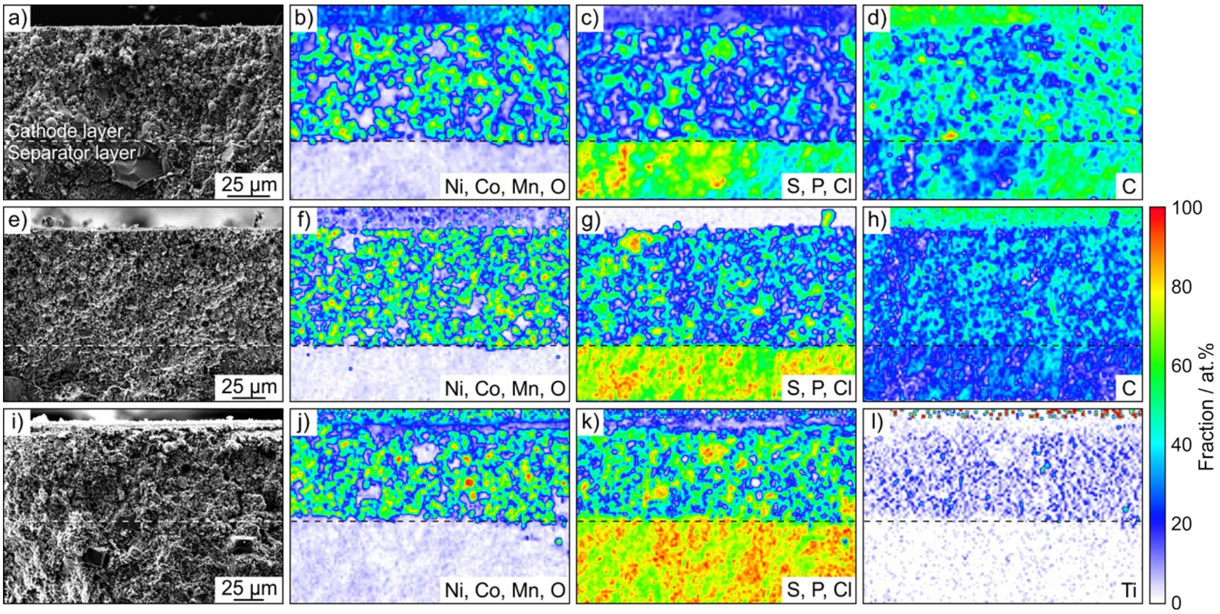

Fig. S4 Cross-sectional SEM images and corresponding elemental maps for the pristine SSB pellet stacks using (a-d) carbon nanofibers, (e-h) Ketjenblack and (i-l) TiC as conductive additive. Only the cathode composite and solid electrolyte separator layers are shown for clarity.

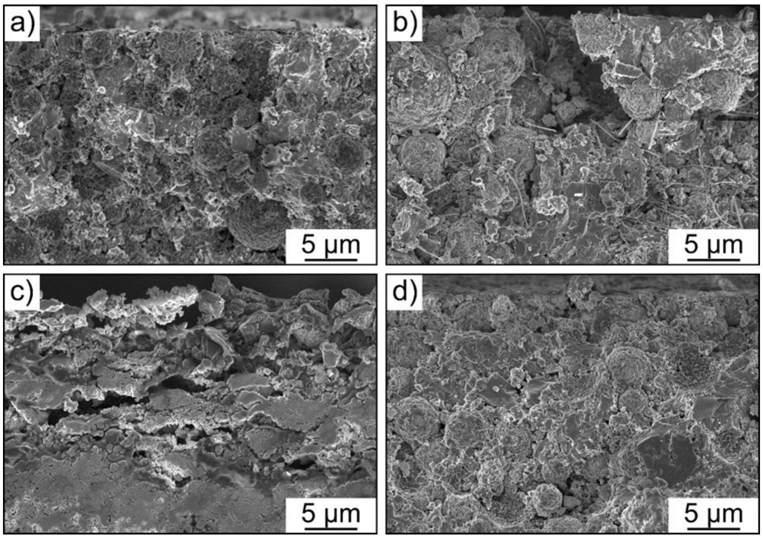

Fig. S5 Cross-sectional SEM images of the cycled SSB pellet stacks using (a) Super C65, (b) carbon nanofibers, (c) Ketjenblack and (d) TiC as conductive additive.

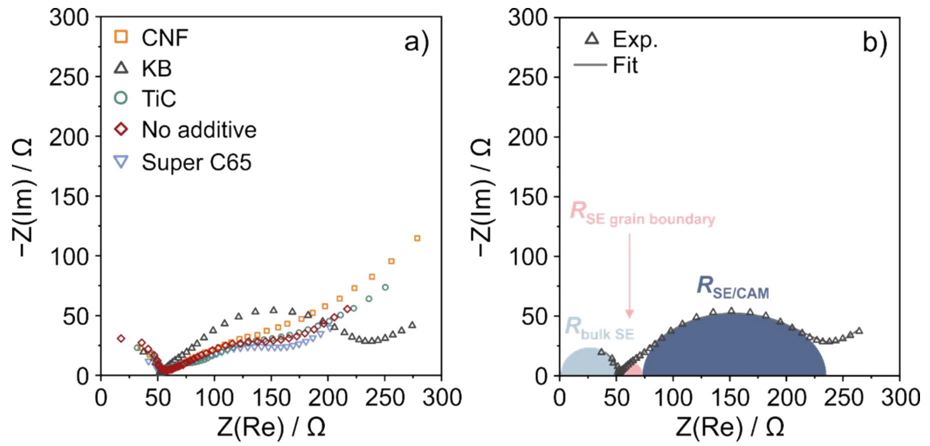

Fig. S6 (a) Room-temperature impedance spectra of the cycled SSB cells with and without conductive additive. (b) Nyquist plot for the cell using Ketjenblack and the corresponding fit to the data. The semicircles shown in (b) are for eye guidance only and represent the resistance contribution from the bulk SE, SE grain boundary and SE/CAM interface.

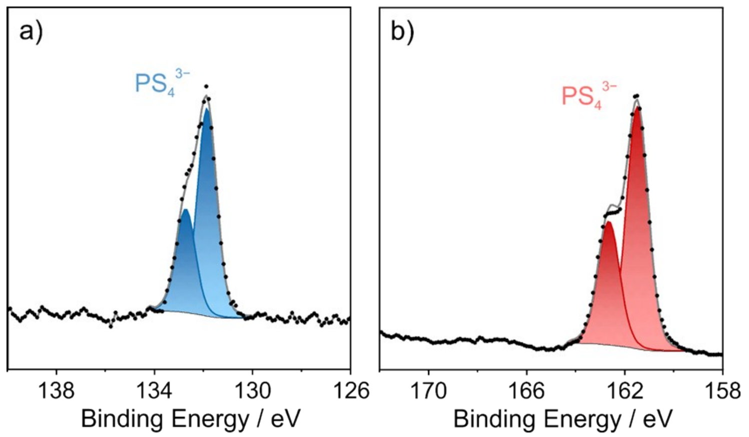

Fig. S7 (a) P 2p and (b) S 2p core-level spectra for the pristine cathode composite using Super C65 as conductive additive. The doublets are characteristic of the PS_4^{3-} unit of argyrodite Li_6PS_5Cl solid electrolyte.

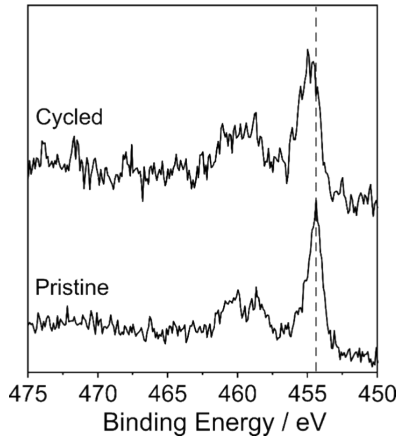

Fig. S8 Ti 2p core-level spectra for the pristine and cycled cathode composites using TiC as conductive additive.
